# Simultaneous display of multiple three-dimensional electrophysiological datasets (dot mapping)

**DOI:** 10.1093/europace/euw190

**Published:** 2016-10-04

**Authors:** Steven E Williams, Nick W F Linton, Steven Niederer, Mark D O'Neill

**Affiliations:** Division of Imaging Sciences and Biomedical Imaging, King's College London, 4th Floor, North Wing, St. Thomas’ Hospital, 249 Westminster Bridge Road, London SE1 7EH, UK

**Keywords:** Electroanatomic mapping, Atrial fibrillation, Ventricular tachycardia, Substrate mapping, Data representation

## Abstract

**Aims:**

Complex ablation procedures are supported by accurate representation of an increasing variety of electrophysiological and imaging data within electroanatomic mapping systems (EMS). This study aims to develop a novel method for representing multiple complementary datasets on a single cardiac chamber model. Validation of the system and its application to both atrial and ventricular arrhythmias is examined.

**Methods and results:**

Dot mapping was conceived to display multiple datasets by utilizing quantitative surface shading to represent one dataset and finely spaced dots to represent others. Dot positions are randomized within triangular (surface meshes) or tetrahedral (volumetric meshes) simplices making the approach directly transferrable to contemporary EMS. Test data representing uniform electrical activation (*n* = 10) and focal scarring (*n* = 10) were used to test dot mapping data perception accuracy. User experience of dot mapping with atrial and ventricular clinical data is evaluated. Dot mapping ensured constant screen dot density for regions of uniform dataset values, regardless of user manipulation of the cardiac chamber. Perception accuracy of dot mapping was equivalent to colour mapping for both propagation direction (1.5 ± 1.8 vs. 4.8 ± 5.3°, *P* = 0.24) and focal source localization (1.1 ± 0.7 vs. 1.4 ± 0.5 mm, *P* = 0.88). User acceptance testing revealed equivalent diagnostic accuracy and display fidelity when compared with colour mapping.

**Conclusion:**

Dot mapping provides the unique ability to display multiple datasets from multiple sources on a single cardiac chamber model. The visual combination of multiple datasets may facilitate interpretation of complex electrophysiological and imaging data.

**What's new?**

- This study develops a novel technology capable of displaying multiple parameters simultaneously on a single cardiac chamber model.
- A combination of colour and monochrome dots allows multiple datasets to be overlaid.
- Examples of both atrial and ventricular datasets are presented for surface and volumetric cardiac chamber models.

## Introduction

Substrate-based ablation strategies underpin the treatment of multiple atrial and ventricular arrhythmias. In identifying arrhythmia substrates, a variety of physiological parameters are proposed, for example late or decrement evoked potentials in ventricular tachycardia (VT)^1,2^ and complex fractionated electrograms (CFE) or low-voltage areas (LVAs) in atrial fibrillation (AF).^3,4^ In certain situations, combinations of datasets become especially valuable, for example bipolar voltage combined with local activation time in macro-re-entrant atrial tachycardia and VT mapping. Furthermore, emerging technologies such as non-invasive body surface mapping^5^ and contact rotor mapping^6^ may provide new physiological information describing the arrhythmia substrate. In addition to these electrical parameters, pre-procedural assessment with atrial and ventricular imaging aims to identify areas of scar,^7^ fibrosis,^8^ or adiposity,^9^ which may be crucial to arrhythmogenesis. Despite this array of complimentary clinical datasets, no contemporary electroanatomic mapping technology is widely available to statically display more than one dataset on a single cardiac chamber model. We therefore aimed to devise a method to represent multiple scalar datasets within a single spatial domain whilst minimizing (1) loss of fidelity in the original datasets and (2) operator-dependent manual segmentation or threshold selection of either dataset. This study demonstrates the application of this system to data representation for both atrial and ventricular arrhythmias.

## Methods

### Dot mapping

**Figure 1:**
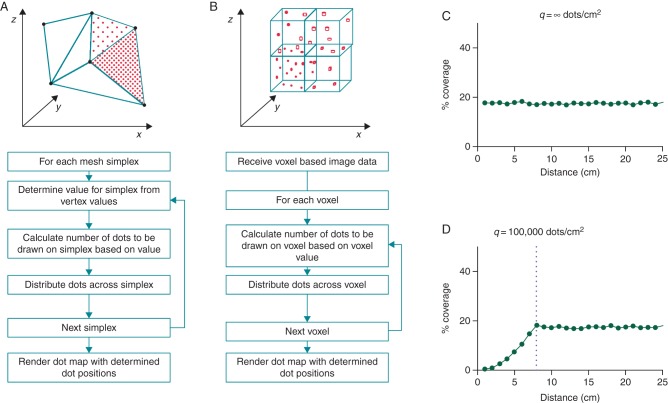
Dot mapping algorithm. (*A*) The dot mapping algorithm as applied to 3D surface shells. (*B*) The dot mapping algorithm as applied to 3D volumetric models. (*C* and *D*) Dot mapping algorithm performance for different values of *q* (see text for details). Distance (cm) represents the distance in the displayed scene between the surface of the cardiac chamber model and the user's viewpoint.

Dot mapping displays multiple datasets on a single cardiac chamber model by representing one dataset with chamber surface shading (colour map), and further datasets by closely spaced dots (dot map) drawn within the same spatial domain. For surface models, dots are drawn over the underlying coloured surface (*Figure 1A*), and for volumetric models, dots are distributed throughout the volume being represented (*Figure 1B*). Dots are related to the dataset in two ways: (i) the location of the dots represents the location of the dataset being represented, and (ii) the density of the dots is proportional to the underlying dataset values. Examples of datasets that might be represented with dot mapping include late gadolinium signal intensity from cardiac magnetic resonance (CMR) imaging or bipolar voltage from electroanatomic mapping. Importantly, since only a fraction of available screen space is used to represent the dataset with dots, it remains possible to ‘see through’ the dots in order to continue to perceive the dataset represented by surface colour shading.

For this proof-of-concept study, the dot mapping algorithms (*Figure 1A* and *B*) were implemented using a commercial software package (MATLAB 8.2, The Mathworks, Inc., MA). Input datasets are represented as either surface or volumetric meshes with associated scalar fields encoding the physiological parameter(s) of interest. For surface shells, point data are converted to element data by taking the mean of the vertex values for each element. Each element within the mesh is assigned a weight (*λ*) based on the volume (or area) of the element (*V*), the scalar value for the element (*S*), and the ratio of the squares of the starting distance between viewpoint and shell (*D*) to the current distance between viewpoint and shell (*D*′):

(1) $$\lambda =\frac{V\times S\times D^{2}}{{D^{\prime }}^{2}}$$

A random number *x* is chosen from the set of positive integers ($k\in N_{1}$) such that the probability of *x*= *k* is given by the Poisson distribution with mean and variance *λ*:

(2) $$P(x=k)=\frac{\lambda^{k}{\text{e}}^{-k}}{k!}$$

In this way, an integer number of dots (*x_i_*) is drawn within each element *i*, based on the scalar value for that element but additionally corrected for element size and viewing position. The coordinates of each dot are calculated using a random spatial distribution within each element. For each triangular element *ABC* (where *A*, *B*, and *C* are vectors defining the *x*, *y*, and *z* Cartesian coordinates of each vertex), this spatial distribution is given by

(3) $$P=(1-\sqrt{r_{1}})A+\sqrt{r_{1}}(1-r_{2})B+\sqrt{r_{1}}r_{2}C$$

where *r*_1_ and *r*_2_ are chosen from a uniform distribution of random numbers in the interval [0,1]. For each tetrahedral element *ABCD*, the spatial distribution is calculated as follows. Let *R* be a three-element array where *r*_1_, *r*_2_, and *r*_3_ are chosen from a uniform distribution of random numbers in the interval [0,1]. *R*′ and *R″* are then defined:

(4) $$R^{\prime }=\left\{\begin{array}{cc} R & \text{if}\;\;r_{1}+r_{2}\leq 1\\ \left[\begin{array}{c} 1-r_{1}\\ 1-r_{2}\\ r_{3} \end{array}\right] & \text{if}\;r_{1}+r_{2}>1\; \end{array}\right.$$

(5) $$R^{\prime \prime }=\left\{\begin{array}{cc} R^{\prime }\; & \text{if}\;r_{1}^{\prime }+r_{2}^{\prime }+r_{3}^{\prime }\leq 1\;\\ \left[\begin{array}{c} r_{1}^{\prime }\\ 1-r_{3}^{\prime }\\ 1-r_{1}^{\prime }-r_{2}^{\prime } \end{array}\right]\; & \text{if}\;r_{1}^{\prime }+r_{2}^{\prime }+r_{3}^{\prime }>1\;\text{and}\;r_{2}^{\prime }+r_{3}^{\prime }>1\;\\ \left[\begin{array}{c} 1-r_{2}^{\prime }-r_{3}^{\prime }\\ r_{2}^{\prime }\\ r_{1}^{\prime }+r_{2}^{\prime }+r_{3}^{\prime }-1 \end{array}\right]\; & \text{if}\;r_{1}^{\prime }+r_{2}^{\prime }+r_{3}^{\prime }>1\;\text{and}\;r_{2}^{\prime }+r_{3}^{\prime }\leq 1\; \end{array}\right.$$

Then, for each dot within an element, the Cartesian coordinates (*P*) are given by

(6) $$P=(1-\Sigma R^{\prime \prime })A+r_{1}^{\prime \prime }B+r_{2}^{\prime \prime }C+r_{3}^{\prime \prime }D$$

The interface allows adjustment of the ‘brightness’ and ‘contrast’ of the dot map by adjusting a constant (*b*), and power (*c*), applied to each element weight (*λ*) to result in a new element weight (*λ*_adj_):

(7) $$\lambda_{\mathrm{adj}}=b\lambda^{c}$$

This new element weight is then used as the mean and variance of the Poisson distribution (Equation 2). Further controls allow the user to toggle display of one or both datasets and perform standard rotate/pan/zoom manipulations. To improve computational efficiency, the number of dots for any one simplex can be limited. If *V* is the volume or area of the simplices and *q* is a system-defined constant, then the maximum number of dots for each simplex (*N*) is given by

(8) N=qA

If the required number of dots (*x_i_*, Equation 2) for a simplex, *i*, is greater than *n_i_*, then the number of dots for that simplex is limited to *n_i_*. The constant *q* is chosen such that ‘dot density’ curves remain flat within expected operational zoom, brightness, and contrast levels.

### Dot perception

To test perception of dual datasets with dot mapping, the following tests were performed: (i) Orientation of the axis of maximal gradient of a scalar field across a two-dimensional (2D) circular geometry (diameter 2 cm) was varied, and the perceived angle of maximal gradient was recorded for colour maps alone, dot maps alone, and combined dot mapping. (ii) Correct localization of the region of the highest scalar field value within a 2D square domain (edge length 2 cm) was determined with colour maps alone, dot maps alone, and combined dot mapping. Example datasets for both experiments are given in Supplementary material online, Figure S1.

### Clinical data collection and pre-processing

#### Atrial cases

Two patients requiring atrial instrumentation were studied. One patient had previously undergone catheter ablation for persistent AF using a stepwise approach incorporating wide-area pulmonary vein encirclement and linear ablation at the mitral isthmus (Case 1). The other patient had previously undergone surgical correction of right and left ventricular outflow tract obstruction obstruction requiring right and left atriotomies. As part of the clinical procedures, a three-dimensional (3D) geometry of the left atrium was created using the Carto 3 mapping system (Biosense Webster, Diamond Bar, CA, USA). High-density, pre-ablation, peak-to-peak, bipolar voltage and activation maps were constructed using points acquired with the ablation catheter to give coverage of the entire chamber of interest. Case 1 underwent CMR imaging 3 weeks prior to ablation, including 3D, balanced, steady-state, free precession (b-SSFP) and 3D late gadolinium enhancement (LGE) imaging. CMR images were processed according to previously described methods.^7^ After semi-automatic segmentation of the atrial blood pool, the LGE images were registered to the b-SSFP images, and a 3D ‘shell’ of the LA was created with LGE data calculated by maximum intensity projection normal to the shell. CMR LGE data were registered to the endocardial electroanatomic mapping (EAM) geometry using landmark registration followed by an iterative closest point algorithm,^10^ which computes an optimal affine-based registration for fusion of the two surfaces.

#### Ventricular cases

A patient requiring left ventricular instrumentation for catheter ablation of ischaemic heart disease-related VT was studied. Endocardial access was obtained using a retrograde aortic approach, and pericardial access was obtained using a subxiphoid percutaneous approach. Using the Carto 3 EAM system, 3D geometries representing the endocardial and epicardial surfaces of the left ventricle were created. High-density, peak-to-peak, bipolar voltage maps were constructed during sinus rhythm. Prior to ablation, the patient underwent CMR imaging including high-resolution 3D LGE imaging. The left ventricular myocardium was segmented using Seg3D (Scientific Computing and Imaging Institute, University of Utah) to create a 3D binary image. A tetrahedral mesh was created from the binary image with mean signal intensity within each tetrahedron represented as the absolute signal intensity value above the mean blood pool value. Finally, the endocardial Carto 3 map was registered to the endocardial surface of the CMR segmentation using landmark registration followed by an iterative closest point algorithm.

### User experience

User experience of dot mapping was assessed in three domains (diagnostic accuracy, dataset fidelity, and ease of use) and compared with traditional display techniques. Each domain was assessed using two atrial paired datasets (electrogram voltage vs. local activation time—see Supplementary material online, *Figure S2*). Users (*n* = 15) reviewed the paired datasets with and without dot mapping for 1 min and, after each 1 min period, the three domains were assessed using a 4-point Likert scale (*Table 1*).

**Table 1:** User experience assessment scale

Domain	Likert items
Diagnostic accuracy	The system maximizes the diagnostic utility of the underlying datasets.
	The system fully facilitates accurate diagnosis.
	The system faithfully represents the first dataset.
	The system faithfully represents the second dataset.
	The system increases the combined value of the underlying datasets.
Display fidelity	The first dataset is displayed with high fidelity.
	The second dataset is displayed with high fidelity.
	The overall fidelity of the system is high.
	There is no loss of fidelity as a result of the display system.
	Registration accuracy between the datasets is maximized.
Ease of use	It is easy to relate the first dataset to the second dataset.
	It is easy to relate the second dataset to the first dataset.
	It is easy to visualize both datasets together.
	The system does not rely on user recollection of the dataset(s).
	The system allows rapid assessment of the combined datasets.

Users rated each Likert item using the 4-point scale: 0, strongly disagree; 1, disagree; 2, agree; 3, strongly agree.

### Statistics

Data analysis was performed using GraphPad Prism version 6.0c (GraphPad Software, San Diego, CA). Continuous variables are expressed as mean ± standard deviation. Groups were compared using the one-way analysis of variance (ANOVA) with Tukey's multiple comparisons test used to identify differences between groups. Likert score results are presented as median scores, and differences were assessed with the Wilcoxon two-sample signed rank test.

## Results

### Dot mapping algorithm performance

Dot mapping ensures that the screen dot density (that is, the number of dots per unit screen area) remains constant for each simplex, or region of simplices with uniform signal intensity, regardless of the zoom level of the scene. In *Figure 1C*, a flat ‘screen dot density’ curve is demonstrated for a region of uniform signal intensity on a 2D test case regardless of zoom level. To improve computational efficiency, the number of dots drawn per unit area can be reduced by adjusting *q* (Equation 8) to *q* < ∞. In doing so, the operational zoom level within which screen dot density is constant is reduced (*Figure 1D*). The dot mapping algorithm allows the ‘brightness’ and ‘contrast’ of the dot map to be adjusted, for example, to avoid areas of saturation at high dataset values or limit the dot density at low dataset values.

### Dataset perception

There was no difference between operator perception accuracy of the orientation of the axis of maximal gradient of a scalar field represented with colour mapping and dot mapping in isolation. In the presence of a second dataset, dot mapping was less accurate than colour mapping, but the absolute error increase was less than 5° (*Table 2*). There was no difference in the localization of a region of high scalar values (e.g. a region of scar) represented with colour mapping or dot mapping either in isolation or in the presence of a second dataset. Finally, the addition of a region of scar represented by dots had no effect on propagation perception accuracy of the underlying isochronal colour map (1.5 ± 1.8° vs. 2.2 ± 2.1°, *P* = 0.45).

**Table 2:** Data perception accuracy

	Single datasets			Combined datasets		
	Colour	Dots	*P*	Colour	Dots	*P*
Maximal gradient direction accuracy (°)	1.5 ± 1.8	4.8 ± 5.3	0.24	2.0 ± 1.2	6.8 ± 5.3	0.047 (*)
Source localization accuracy (mm)	1.1 ± 0.7	1.4 ± 0.5	0.88	1.8 ± 0.6	2.6 ± 0.9	0.076

Maximal gradient direction accuracy (degrees) and source localization accuracy (mm) were assessed for colour mapping vs. dot mapping in isolation (‘single datasets’) and colour mapping vs. dot mapping in the presence of a second dataset (i.e. dots or colour, ‘combined datasets’). *, *P* < 0.05.

### Clinical cases

#### Right atrial electrogram voltage with local activation time

**Figure 2:**
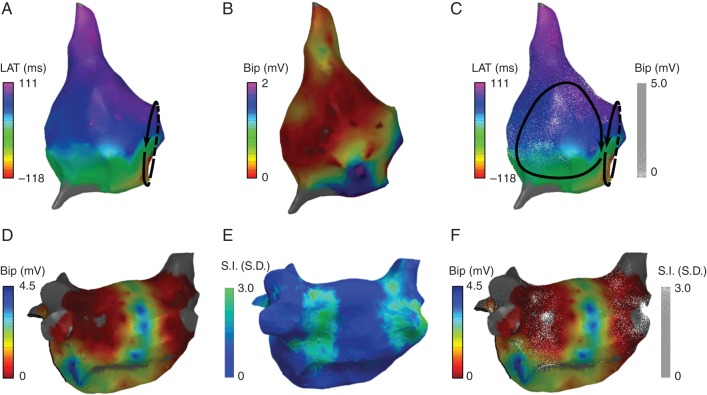
Dot mapping atrial cases. (*A* and *B*) Right atrial maps showing local activation time and bipolar electrogram voltage, respectively. (*C*) Combined dot map showing local activation time (colour isochrones) and bipolar voltage (dots). (*D* and *E*) Post-ablation left atrial maps showing bipolar electrogram voltage and CMR LGE signal intensity, respectively. (*F*) Combined dot map showing bipolar electrogram voltage (colour) and CMR LGE signal intensity (dots).

*Figure 2A–C* shows an example of dot mapping representing electroanatomic mapping data. In (*A*), a traditional isochronal activation map created by point-by-point mapping of the right atrium is shown. The activation pattern is consistent with typical right atrial flutter. In (*B*), a voltage map is shown with a threshold of 0.5 mV applied, revealing a lateral scar (purple) consistent with the previous atriotomy. In (*C*), the combined display of voltage and activation data alerts the operator to a possible second circuit involving the region of scar in the lateral right atrium. Entrainment in this case confirmed that this was in fact the primary tachycardia circuit.

#### Left atrial electrogram voltage and CMR late gadolinium enhancement

Dot mapping can be used to combine datasets from multiple modalities. In *Figure 2D–F*, a left atrial chamber model from a patient with post-ablation atrial tachycardia is shown. In (*D*), the EAM bipolar voltage map created in sinus rhythm demonstrated clearly delineated zones of low voltage around the pulmonary veins. In (*E*), the LGE shell shows enhancement (green) indicative of post-ablation atrial scar. The combination of both datasets in (*F*) using dot mapping allows the qualitative relationship between scar and voltage to be perceived. In particular, the density of the scar can be perceived with dot density reflecting the underlying signal intensity, i.e. the greater the dot density, the higher the LGE signal intensity.

#### Ventricular endocardial and epicardial voltage maps

**Figure 3:**
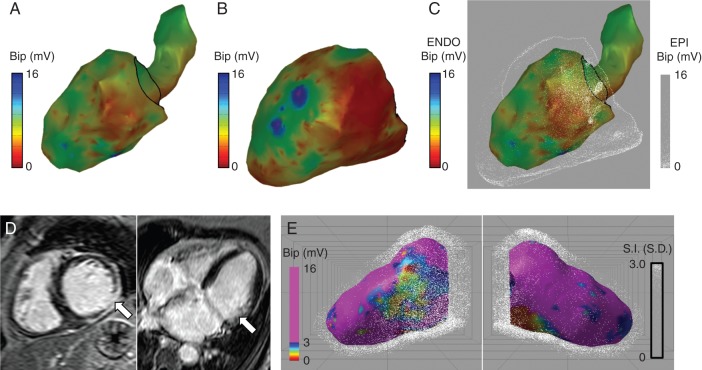
Dot mapping ventricular cases. (*A* and *B*) Left ventricular bipolar electrogram voltage maps for endocardial and epicardial shells, respectively. (*C*) Combined left ventricular bipolar electrogram voltage maps showing endocardial voltage (colour) and epicardial voltage (dots). (*D*) CMR LGE showing scar in the mid to basal inferolateral wall. (*E*) Combined dot map showing bipolar electrogram voltage (colour) and CMR LGE (dots).

In the atrial cases, above the dot map is drawn a small distance above the shell surface. Extending this approach can allow the dot map to be drawn some distance from the surface of the underlying colour map allowing the technique to be applied to ventricular endocardial and epicardial mapping. In *Figure 3C*, combined endocardial and epicardial voltage maps are shown using dot mapping. Compared with the individual colour shells, dot mapping (*C*) allows continued perception of the endocardial voltage map even when it is overlaid with the epicardial map. Furthermore, by manipulating the orientation of the dot map, it becomes possible to perceive the relationship between endocardial scar and epicardial scar (see Supplementary material online, *Video S1*).

#### Ventricular voltage mapping and late gadolinium enhancement cardiac magnetic resonance

Dot mapping accepts volumetric datasets in addition to surface shells. In *Figure 3D* and *E*, the same case as above is displayed using dot mapping to display a combination of endocardial voltage and LGE CMR. The pre-ablation CMR scan in this patient identified a 75% thickness subendocardial scar in the mid to basal inferolateral wall (white arrows, *D*). By representing the CMR data with volumetric dot mapping, it becomes possible to perceive the relationship between the ischaemic scar and endocardial low-voltage regions.

### User experience

With 5 items in each domain, the maximum score per domain is 25. Dot mapping outperformed colour mapping in the ‘dataset co-registration’ domain (median score 24.5 vs. 10, *P* < 0.05). ‘Diagnostic accuracy’ (17 vs. 21) and ‘display fidelity’ (18 vs. 20) scored similarly, indicating that dot mapping faithfully represents the input datasets and is non-inferior to the separate display of the individual datasets.

## Discussion

The main findings of this study are as follows. (i) Dot mapping is a new technique that can display at least two independent datasets within a common spatial domain whilst minimizing loss of fidelity of either dataset. (ii) Dot mapping can be applied to electrophysiological and imaging datasets in both the atrium and the ventricle. (iii) Dot mapping can facilitate the clinical interpretation of a second dataset in the context of a first dataset.

### Motivation

Three developments over the past two decades promoted the design of the dot mapping system. Firstly, the quantity of data available to support clinical diagnoses of arrhythmia mechanisms in electrophysiology procedures is increasing. In early ablation procedures, activation times at a limited number of electrodes were used to diagnose the arrhythmia and demonstrate successful application of radiofrequency (RF) energy,^11^ but in modern procedures, mapping may be achieved using catheters with tens of electrodes recording hundreds of data points. The corresponding increase in the quantity of clinical data has led to techniques to summarize this data, reducing the number of dimensions involved and facilitating its display on 3D representations of a cardiac chamber. Secondly, with greater geometrical position and electrical accuracy of mapping systems, the opportunity has arisen to interpret electrograms in an increasing number of ways, including amplitude (voltage), timing (local activation time), complexity (fractionation), and under an increasing number of conditions (e.g. sinus rhythm, during tachyarrhythmia, and during pacing manoeuvres). Finally, an increased understanding of arrhythmia mechanisms requires that combinations of these parameters are considered simultaneously (e.g. voltage or imaging-derived scar and local activation time) in order to reach a clinical diagnosis.

### Relevance to prior work

A limited number of techniques have previously been used to facilitate the co-display of more than one dataset across a spatial domain. Some of these are specific to electrophysiological procedures, whilst some are not. They include surface transparency, linked scenes, and ripple mapping.^12^ All of these have disadvantages for the co-display of scalar fields in electrophysiology. The use of surface transparency does allow simultaneous colouring of a shell to represent two datasets, but combinations of colours result in confusion for interpretation, and in practice, this technique limits the number of colours that can be used in one or both datasets. Displaying linked scenes, with two copies of the same geometry coloured differently and either side by side or toggled on user selection, relies on greater geometrical perception by the operator. Finally, in ripple mapping, neither of these limitations applies, but the system specifically represents electrogram voltage over time along with one other dataset and requires the display to be played as a moving scene, the spatial perception of which requires habituation to interpret the data.

Considering the requirement for multiple dataset display in electrophysiological procedures and the aforementioned limitations of the existing techniques, this study developed a system that could display at least two independent scalar fields across the surface of a single cardiac chamber shell. One dataset is represented using a colour field, whilst the second dataset is represented using finely spaced dots of uniform colour. In doing so, perception of the datasets depends on the entirely different visual inputs of colour or quantity. The fact that dots are displayed as single pixels with a limited maximum density ensures that the underlying colour data set can still be perceived.

### Clinical applications

This paper presents a proof-of-concept study of a methodology for overlaying multiple datasets in electroanatomic mapping platforms, which should undergo further clinical evaluation. Potential applications of dot mapping include representation of both electrogram- and imaging-derived datasets, in particular where combinations of these datasets could yield additional clinically relevant diagnostic information. As a research tool, dot mapping could allow pre-procedural measurements, for example, from electrocardiographic^13^ or CMR imaging^14^ to be directly compared with intra-procedural measurements, for example electrogram complexity or voltage.^7^ As a clinical tool, dot mapping could facilitate co-representation of datasets in both atrial and ventricular ablation procedures. A particular strength of dot mapping may be the interpretation of activation maps in scar-related atrial tachycardia.^15^ During AF ablation procedures, both LVAs^3^ and regions of signal complexity^16^ have been used as targets for substrate modification. By representing the intersection of LVAs and regions of signal complexity, dot mapping may reveal atrial regions of particular importance to arrhythmogenesis. Finally, advanced imaging techniques may soon aid identification of atrial or ventricular regions (e.g. the critical isthmus in VT) crucial to arrhythmogenesis.^17^ Dot mapping would allow this information to be available to the operator without hindering intra-procedural collection or display of electroanatomical mapping data in the usual way.

### Limitations

Perception of datasets using dot mapping relies on the proximity between finely spaced dots. In order to correctly perceive this relationship, the viewing position should be normal to the region of interest. Since complex chamber geometries are viewed in electroanatomic mapping platforms, the perception of dot mapping is likely to require additional map manipulation compared with colour mapping.

## Conclusions

This study has developed a new technology that facilitates the display and clinical interpretation of multiple electrophysiological datasets. We have demonstrated the use of the system with both atrial and ventricular data using both surface and volumetric chamber models. Future work should assess intra-procedural use of dot mapping in the management of complex arrhythmias.

## Funding

This work was supported by the National Institute for Health Research (NIHR) Biomedical Research Centre at Guy's and St Thomas’ NHS Foundation Trust and King's College London, and the British Heart Foundation (PG/13/37/30280). The views expressed here are those of the authors and not necessarily those of the NHS, the NIHR, or the Department of Health.

**Conflicts of interest:** The work presented in this manuscript is subject to a patent application: International Patent Application No. PCT/GB2014/050603 with publication number WO 2014/132080. Method and System for Overlaying Data or Images. Williams and Linton; King's College London.

## Supplementary Material

Supplementary Figure S1

Supplementary Figure S2

Supplementary Video S1
