# Supplementary figures and images for: Simultaneous display of multiple three-dimensional electrophysiological datasets (dot mapping)
Source: Europace. 2016 Oct 4;19(10):1743–9. doi: 10.1093/europace/euw190 (PMC5834094; doi:10.1093/europace/euw190)

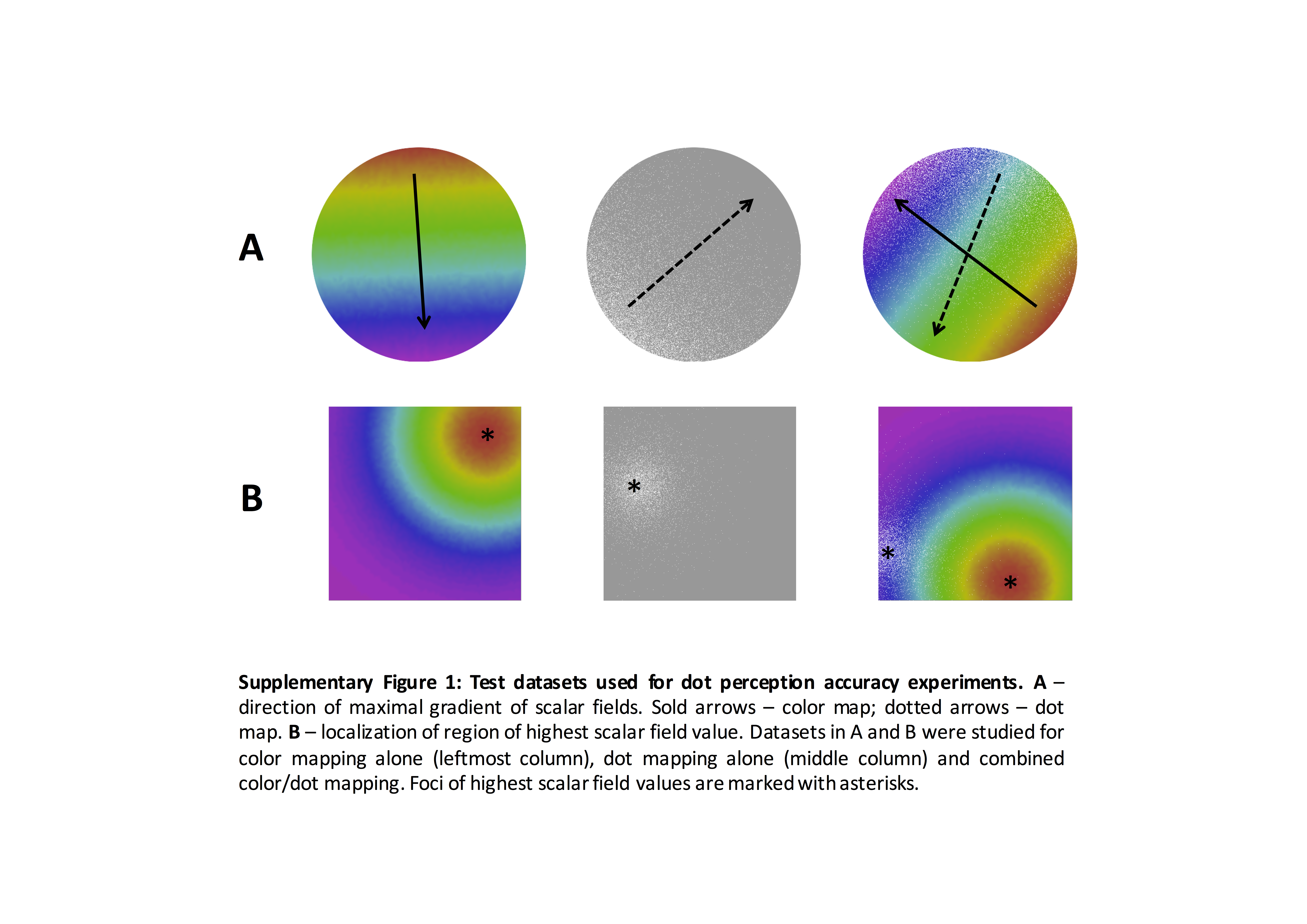

Supplement: Supplementary Figure S1 [file supplementary_figure_1_euw190.png]

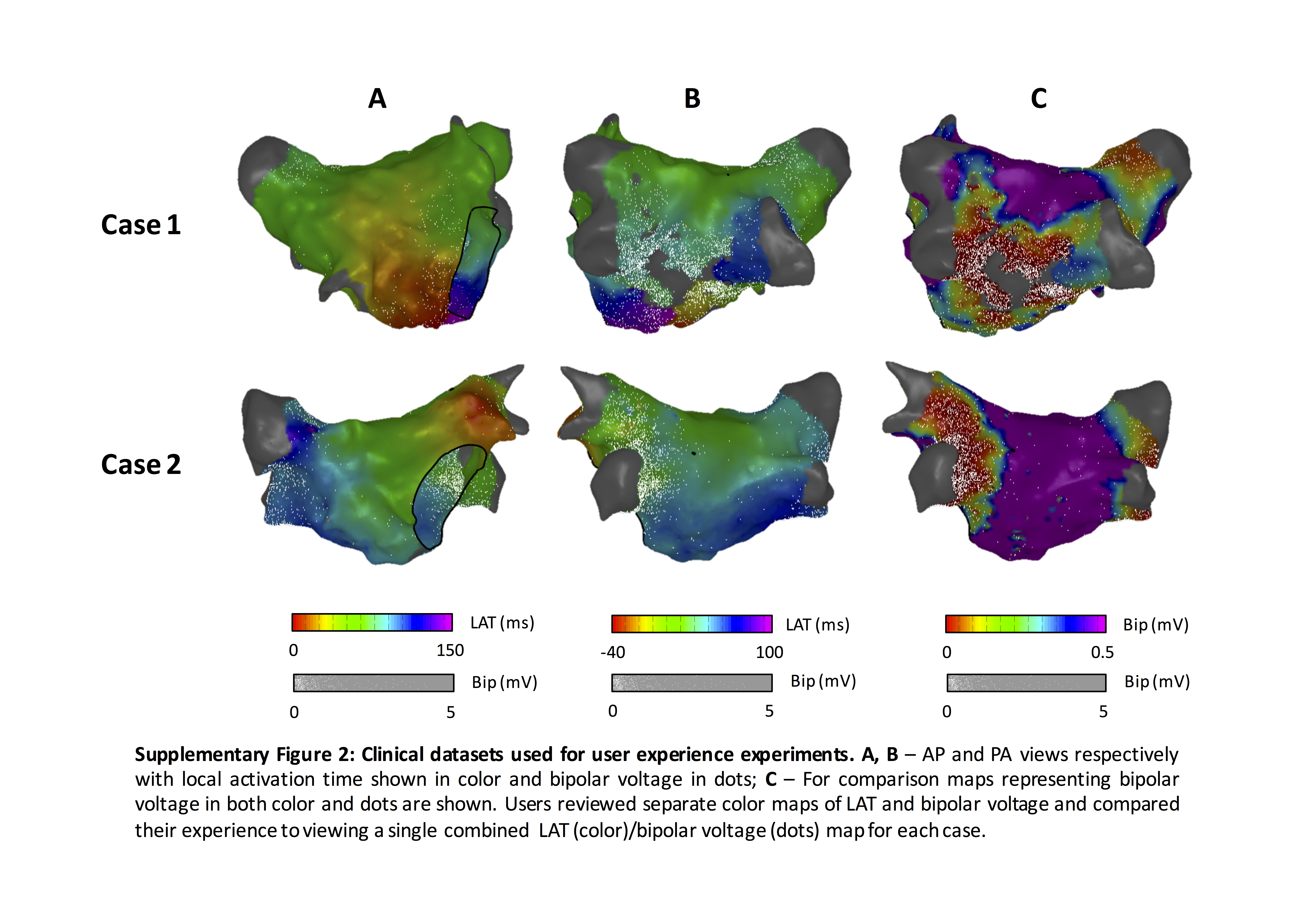

Supplement: Supplementary Figure S2 [file supplementary_figure_2_euw190.png]
